# A dataset of Chinese drivers’ driving behaviors and socio-cultural factors related to driving

**DOI:** 10.1016/j.dib.2023.109337

**Published:** 2023-06-22

**Authors:** Ruining Jin, Xiao Wang, Minh-Hoang Nguyen, Viet-Phuong La, Tam-Tri Le, Quan-Hoang Vuong

**Affiliations:** a Civil, Commercial and Economic Law School, China University of Political Science and Law, Bei-jing, 100088, China; b Suzhou Lunhua Education Group, Suzhou, China; c Centre for Interdisciplinary Social Research, Phenikaa University, Yen Nghia Ward, Ha Dong District, Hanoi 100803, Vietnam; d A.I. for Social Data Lab (AISDL), Vuong & Associates, Hanoi 100000, Vietnam

**Keywords:** Aggressive driving behaviors, Safe driving behaviors, Friends, Peers, Family, Mindsponge theory

## Abstract

Given the high fatality rate due to road traffic accidents in China, understanding the factors influencing aggressive driving behaviors among Chinese drivers is essential to alleviate the problem. The paper describes a dataset of 1039 Chinese drivers’ driving behaviors and the socio-cultural factors associated with the behaviors. The dataset was collected through an online survey. The dataset comprises five main categories: 1) driving information, 2) aggressive driving behaviors, 3) friend/peer influence, 4) family influence, and 5) socio-demographic information. The dataset is valuable for public health and transportation researchers to explore factors influencing drivers’ driving behaviors and public safety in China. The dataset's construct validity was confirmed by the Bayesian Mindsponge Framework (BMF) analytics. Specifically, the analysis shows that safe driving behaviors are affected by information promoting safe driving that is passively and actively absorbed from friends/peers (friends/peers being role models and friends’/peers’ support, respectively). The result is consistent with the Mindsponge Theory's information-processing mechanism in human minds.

**Specifications Table**

**Table:** 

Subject	• Public health • Social sciences
Specific subject area	• Public health and health policy • Social sciences • Psychology
Type of data	• Survey data
How the data were acquired	Initially, online interviews were conducted with 54 participants to identify their aggressive driving behaviors and understand the reasons behind those behaviors. All the interviews were conducted through WeChat from April 10 to 25, 2022. Then, convenient sampling was employed for survey collection. The survey was designed on the WeChat mini-app “Survey Star.” The group owners distributed the link to the survey in four WeChat public groups from May 1 to 5, 2022. It is important to note that all group members were insured by the company owned by the group owners. These WeChat public groups were selected to ensure all the respondents own a vehicle. Each group has 500 members at its maximum, which culminated in a total of 2000 drivers. In total, 1039 drivers completed the online survey. A response rate of 51.95% was obtained.
Data format	Raw, Analyzed and/or Filtered
Description of data collection	The dataset consists of responses from 1039 Chinese respondents. Most respondents were males, accounting for 61.50%. The educational levels of the respondents were distributed relatively equally across the middle school and below (17.81%), high school or vocational college (29.45%), undergraduate or associate college (28.49%), master's degree (20.60%), and doctoral degree (3.66%). Most respondents had a monthly income of 3000 to 15000 RMB (69.78%). People in the age group of 25-40 occupied the highest percentage of the respondents. Around 90% of the respondents sometimes/often/always drove their vehicles for work. Most respondents had 1-3 years of holding a driving license (39.27%). Meanwhile, 17.81% of the respondents had a driving license for less than one year, and 6.16% had a driving license for more than ten years.
Data source location	• Country: China
Data accessibility	The dataset (saved and encoded in a CSV file) and its detailed description (saved in an XLSX file) are deposited together at: https://osf.io/stcj2/ (DOI:10.17605/OSF.IO/STCJ2)

## Value of the Data

• The dataset offers the resources to study the influences of socio-cultural elements on Chinese drivers’ aggressive driving behaviors.
• Researchers in the public health and transportation fields can employ the current dataset to generate insightful results for reducing traffic accidents in China.
• Socio-cultural researchers can employ the dataset to explore the influences of socio-cultural factors on Chinese driving behaviors.
• Making the data open helps reduce the costs of conducting public health and transportation research on road traffic accidents, increases transparency and integrity, and supports reproducibility in the future.

## 1 Objective

According to the World Health Organization (WHO), road traffic accident was the 8^th^ leading cause of death in 2019, with 17.38 deaths per 100,000 population [1]. Besides the health costs, road traffic incidents also incurred an accumulative economic loss of over 352 billion yuan from 1996 to 2015 [2]. Risky and aggressive driving behavior is one of the reasons behind the high fatality of road injury in China [2]. Thus, studying the factors influencing drivers’ aggressive driving behaviors is imperative. Many studies have been conducted to reveal the predictors of aggressive driving behaviors, such as drivers’ socio-demographic attributes, personality, seasonal variations, the period of day (peak or off-peak), the type of highway, and characteristics of the built environment [3], [4], [5], [6], [7], [8], [9]. However, the knowledge of socio-cultural influences on these behaviors remains limited. The current dataset offers public health and transportation researchers the resources to discover the influences of socio-cultural factors on Chinese drivers’ aggressive driving behaviors. Also, making the data open helps reduce the costs of conducting public health and transportation research on road traffic accidents, increases transparency and integrity, and supports reproducibility in the future [10,11].

## 2 Data Description

### 2.1 Data sample

The dataset consists of responses from 1039 Chinese respondents. Most respondents were males, accounting for 61.50%. The educational levels of the respondents were distributed relatively equally across the middle school and below (17.81%), high school or vocational college (29.45%), undergraduate or associate college (28.49%), master's degree (20.60%), and doctoral degree (3.66%). Most respondents had a monthly income of 3000 to 15000 RMB (69.78%). People in the age group of 25-40 occupied the highest percentage of the respondents, with 65.54%, whereas only 2.60% of the respondents belonged to the age group of 50+.

Around 90% of the respondents sometimes/often/always drove their vehicles for work. Most of the respondents had 1-3 years of holding a driving license (39.27%). Meanwhile, 17.81% of the respondents had a driving license for less than one year, and 6.16% had a driving license for more than ten years.

### 2.2 Response coding

The current section presents how the responses of five major categories were coded according to the following order:

1) Driving information,
2) Aggressive driving behaviors,
3) Friend/peer influence,
4) Family influence, and
5) Socio-demographic information.

Two main types of responses are categorical (including binary variables) and numerical variables. In the following subsections, we describe categorical variables using seven kinds of information corresponding with seven columns: “Variable,” “Name,” “Explanation,” “Level,” “Code,” “Frequency,” and “Proportion.” For numerical variables, the last three columns are replaced with “Range,” “Mean,” and “Standard deviation.”

#### 2.2.1 Driving information

The dataset's first category comprises five variables (three categorical and two numerical variables). These variables were generated from questions about drivers’ driving experience, skill, and confidence. They are coded as *A1* to *A5* (Table 1).

**Table 1:** Description of variables related to driving information.

Categorical variables						
Variable	Name	Explanation	Level	Code	Frequency	Proportion
*A1*	Commercial insurance for the vehicle	Whether the respondents have commercial insurance for their vehicles	Yes	1	1039	100.00%
			No	0	0	0.00%

*A2*	Frequency of driving to work	How often the respondents drive to work	Never	1	1	0.10%
			Rarely	2	120	11.55%
			Sometimes	3	475	45.72%
			Often	4	310	29.84%
			Always	5	133	12.80%

*A5*	Length of time having a driver's license	How long the respondents have had a driver's license	Less than 1 year	1	185	17.81%
			1-3 years	2	408	39.27%
			3-5 years	3	257	24.74%
			5-10 years	4	125	12.03%
			More than 10 years	5	64	6.16%

Numerical variables						

Variable	Name	Explanation	Level	Range	Mean	Standard deviation

*A3*	Confidence in driving skills	The confidence level of respondents in their driving skills	1. Not at all confident 2. Not very confident 3. Confident 4. fairly confident 5. Very confident	1-5	3.38	0.76

*A4*	Concern about consequences of negligent driving	The concern level of respondents about the consequences of negligent driving	1. Not at all concerned 2. Not very concerned 3. Concerned 4. fairly concerned 5. Very confident	1-5	3.34	0.75

#### 2.2.2 Aggressive driving behaviors

The second category focuses on the drivers’ aggressive driving behaviors (see Table 2). The category consists of seven numerical variables, coded as *B1* to *B7*. The questions used to generate these variables were generated by referring to the Aggressive Driving Behavior Scale and modification based on the former interview with 54 Chinese drivers.

**Table 2:** Description of variables related to aggressive driving behaviors.

Numerical variables						
Variable	Name	Explanation	Level	Range	Mean	Standard deviation
*B1*	Speed limit: rarely exceed	Agreement with the following statement [I rarely exceed the speed limit while driving]	1. Strongly disagree 2. Disagree 3. Neutral 4. Agree 5. Strongly agree	1-5	3.82	1.25
*B2*	Normal speed, avoid weaving, reckless overtaking	Agreement with the following statement [I try to maintain a normal speed while driving and avoid weaving through traffic or overtaking recklessly]			3.80	1.28
*B3*	Safe distance, no tailgating	Agreement with the following statement [Even if other drivers are driving too slowly, I maintain a safe distance and do not tailgate]			3.78	1.27
*B4*	No retaliation: flashing high beams, changing lanes abruptly	Agreement with the following statement [I don't retaliate by flashing high beams when other drivers drive too slowly or forget to turn off their high beams]			3.72	1.29
*B5*	Use turn signal, give heads-up, don't change lanes abruptly	Agreement with the following statement [I don't change lanes abruptly when the traffic flow on my lane is slow. If I do need to change lanes, I use my turn signal and give a heads-up to the drivers behind me]			3.80	1.26
*B6*	Allow other drivers to merge	Agreement with the following statement [I allow other drivers to merge into my lane]			3.78	1.27
*B7*	Slow down, stop at yellow light	Agreement with the following statement [I always slow down and stop when I see a yellow light instead of accelerating through it]			3.78	1.25

#### 2.2.3 Friend/peer influence

The third category focuses on the influence of drivers’ peers/friends on their driving behaviors (see Table 3). This category has nine variables, reflecting peers’/friends’ support for safe driving (variables *C1*-*C6*), peers/friends being role models (variables *C7* and *C8*), and drivers’ care for their peers’/friends’ safety when being in the car (variable *C9*).

**Table 3:** Description of variables related to friend/peer influence.

Numerical variables						
Variable	Name	Explanation	Level	Range	Mean	Standard deviation
*C1*	Supportive friends	Agreement with the following statement [My friends support me in safe driving]	1. Strongly disagree 2. Disagree 3. Neutral 4. Agree 5. Strongly agree	1-5	3.82	1.27
*C2*	Advocating safe driving	Agreement with the following statement [My friends always advocate for safe driving even when in a hurry]			3.80	1.24
*C3*	Caution against driving under the influence	Agreement with the following statement [My friends caution me against driving under the influence of alcohol or drugs]			3.76	1.23
*C4*	Respect for decision not to drink/use drugs before driving	Agreement with the following statement [My friends respect my decision not to drink or use drugs before driving]			3.80	1.24
*C5*	Commendation for slowing down at yellow lights	Agreement with the following statement [My friends commend me for slowing down at yellow lights]			3.78	1.26
*C6*	Praise for courteous and yielding behavior	Agreement with the following statement [My friends praise instead of ridicule me for being courteous and yielding to other vehicles]			3.78	1.26
*C7*	No drinking/drug use while driving	Agreement with the following statement [I have never seen my friends drink or use drugs and drive]			3.82	1.23
*C8*	Rarely witness uncivilized driving behaviors	Agreement with the following statement [I rarely witness my friends engaging in uncivilized driving behaviors such as reckless lane changes, speeding, or flashing]			3.79	1.24
*C9*	Increased caution with friends in car	Agreement with the following statement [I am more careful while driving when my friends are in the car]			3.74	1.26

#### 2.2.4 Family influence

Family influence on drivers’ driving behaviors can be studied using variables in the fourth category (see Table 4). This category has 12 variables, reflecting how family members influence the drivers’ driving behaviors.

**Table 4:** Description of variables related to family influence.

Numerical variables						
Variable	Name	Explanation	Level	Range	Mean	Standard deviation
*D1*	Planned driving	Agreement with the following statement [My parents are good at planning ahead so that they don't have time pressure when driving]	1. Strongly disagree 2. Disagree 3. Neutral 4. Agree 5. Strongly agree	1-5	3.79	1.26
*D2*	Follow traffic rules	Agreement with the following statement [Even when in a hurry, my parents follow traffic rules]			3.80	1.23
*D3*	Praise safe driving	Agreement with the following statement [My parents praise me for safe driving]			3.79	1.27
*D4*	Criticize unsafe driving	Agreement with the following statement [My parents criticize me for unsafe driving]			3.75	1.26
*D5*	Discuss safe driving importance	Agreement with the following statement [My parents talk to me about safe driving and its importance]			3.81	1.25
*D6*	Share driving experiences	Agreement with the following statement [My parents share their own safe driving experiences and lessons with me]			3.81	1.25
*D7*	Monitor driving behavior	Agreement with the following statement [My parents monitor my driving habits and behavior]			3.75	1.25
*D8*	Committed to safe driving	Agreement with the following statement [My parents are committed to safe driving and its values]			3.77	1.27
*D9*	Set example for safe driving	Agreement with the following statement [My parents set an example of safe driving for me and others]			3.79	1.25
*D10*	Give clear rules for safe driving	Agreement with the following statement [My parents give me clear information and rules for safe driving]			3.76	1.23
*D11*	Limit driving at night or bad weather	Agreement with the following statement [My parents limit my driving at night or in bad weather]			3.77	1.24
*D12*	Drive more carefully with family	Agreement with the following statement [I drive more carefully when my parents, children, or spouse are in the car with me]			3.81	1.24

#### 2.2.5 Socio-demographic information

The socio-demographic information of drivers is recorded by variables in the fifth category (see Table 5). It includes the drivers’ self-identified gender (*E1*), educational level (*E2*), monthly income (*E3*), and age (*E4*).

**Table 5:** Description of variables related to socio-demographic information.

Categorical variables						
Variable	Name	Explanation	Level	Code	Frequency	Proportion
*E1*	Gender	Self-identified gender	Male	1	639	61.50%
			Female	2	400	38.50%
			Other	3	0	0.00%
*E2*	Education level	Educational level	Middle school or below	1	185	17.81%
			High school or vocational college	2	306	29.45%
			Undergraduate or associate degree	3	296	28.49%
			Master's degree	4	214	20.60%
			Doctoral degree	5	38	3.66%
*E3*	Monthly income	Monthly income (in RMB)	Below 3000	1	145	13.96%
			3000-9000	2	385	37.05%
			9000-15000	3	340	32.72%
			15000-30000	4	142	13.67%
			Above 30000	5	27	2.60%
*E4*	Age	Age range	18-25	1	161	15.50%
			25-30	2	285	27.43%
			30-40	3	396	38.11%
			40-50	4	170	16.36%
			50+	5	27	2.60%

## 3 Experimental Design, Materials and Methods

### 3.1 Survey design and collection procedure

The survey was systematically designed with five major steps: (1) Pilot interviews and literature review, (2) questionnaire design, (3) survey collection, (4) dataset generation, and (5) data analysis.

Initially, online interviews were conducted with 54 participants to identify their aggressive driving behaviors and understand the reasons behind those behaviors. Specifically, the interviewees were asked how their peers/friends and parents influenced their driving behaviors. All the interviews were conducted through WeChat from April 10 to 25, 2022. These participants were members of a WeChat public group of drivers who had purchased insurance from the same insurance provider that established the group. Each interview lasted approximately 15-30 minutes. The interview results were later used to formulate questions in the questionnaire.

In addition, the Aggressive Driving Behavior Scale was employed to measure the drivers’ self-rated aggressive driving behavior. However, based on the interviews’ results, some of the questions in the scale were modified and localized to make them more appropriate to the Chinese context. In particular, the original Aggressive Driving Behavior Scale is an 11-item questionnaire with a 6-point Likert scale (1 – never, 2 – almost never, 3 – sometimes, 4 – fairly often, 5 – very often, 6 – always) [12]. The localized version used a 5-point Likert scale (from 1 – highly disagree to 5 – highly agree), condensed 11 items to 7 items, and employed a positive tune to lower participants’ resistance to questions that might sound offensive and face-losing in Chinese culture.

Then, convenient sampling was employed for survey collection. The survey was designed on the WeChat mini-app “Survey Star.” The group owners distributed the link to the survey in four WeChat public groups from May 1 to 5, 2022. It is important to note that all group members were insured by the company owned by the group owners. These WeChat public groups were selected to ensure all the respondents own a vehicle. Each group has 500 members at its maximum, which culminated in a total of 2000 drivers. In total, 1039 drivers completed the online survey. A response rate of 51.95% was obtained. All participants received informed consent before they started filling in the questionnaire. All the samples are saved and encoded in a CSV file for later analysis. The detailed description of the dataset is shown in an XLSX file which is deposited together with the dataset at: https://osf.io/stcj2/

## 4 Dataset Validation

We employed the Bayesian Mindsponge Framework (BMF) analytics to check the construct validity of the dataset [13,14]. BMF analytics utilizes the Mindsponge Theory for theoretical reasoning and Bayesian inference for statistical analysis [15], [16], [17]. Specifically, we conducted the Bayesian analysis to test the hypothesized relations based on the information-processing mechanism of Mindsponge Theory. The Mindsponge Theory has effectively explained various complex socio-psychological phenomena [18,19]. Thus, the dataset is deemed valid if the findings generated using the current dataset are consistent with the Mindsponge Theory.

In the information-processing mechanism of the Mindsponge Theory, information is considered as the foundation on which physical reality is constructed, so the social interactions can be viewed as the information-exchange processes among minds (or information collection-cum-processors). The information-processing mechanism is obliged to the set theory logic, so a psychological or behavioral phenomenon can be measured based on the existence and density of the information within a conceptual set (i.e., mind, environment, social interactions). For example, *C7* and *C8* variables help measure the accessibility and the accessible information density of a respondent to safe driving information from their friends, who are regarded as information sources.

One of the fundamental assumptions of the Mindsponge Theory is that the human mind tends to be influenced by the information absorbed from trusted external sources. To test this assumption, we examined whether safe driving behaviors are affected by information promoting safe driving that is absorbed from friends. To measure the safe driving behaviors of the respondents, we created the composite variable *SafeDriving* from averaging variables *B1* to *B7*. The internal reliability of these seven variables is acceptable, with a Cronbach's alpha being 0.943.

Friends are selected as external information sources because respondents tend to trust people they consider friends. There are two ways information promoting safe driving can be absorbed from friends: active and passive absorption. Active absorption is measured by questions in the sub-category “Friends’ role model,” which reflects the degree the respondents observe the safe driving behaviors of their friends. *FriendRoleModel* is the composite variable generated by averaging variables *C7* and *C8*. The internal reliability of these two variables is acceptable, with a Cronbach's alpha being 0.826 [20].

Meanwhile, passive absorption is measured by questions in the sub-category “Friends’ support,” which reflects the degree to which the respondents were supported to drive safely by friends. *FriendsSupport* is the composite variable generated by averaging variables *C1* to *C6*. The internal reliability of these six variables is acceptable, with a Cronbach's alpha being 0.933.

In general, we tested the following model:

(1.1) SafeDriving∼normal(μ,σ)

(1.2) $$\mu_{i}=\beta_{0}+\beta_{FriendRoleModel\;}*FriendRoleModel{\;}_{i}+\beta_{FriendsSupport\;}*FriendsSupport{\;}_{i}$$

(1.3) β∼normal(M,S)

The probability around μ is determined by the form of the normal distribution, whose width is specified by the standard deviation σ. $\mu_{i}$ indicates the respondent i’s degree of safe driving behavior; $FriendRoleModel{\;}_{i}$ indicates respondent i’s degree of absorbing information promoting safe driving from friends actively; $FriendsSupport{\;}_{i}$ indicates respondent i’s degree of absorbing information promoting safe driving from friends passively. Model 1 has four parameters: the coefficients, $\beta_{FriendRoleModel\;}$ and $\beta_{FriendsSupport\;}$, the intercept, $\beta_{0}$, and the standard deviation of the “noise”, σ. The coefficients of the predictor variables are distributed as a normal distribution around the mean denoted M and with the standard deviation denoted S.

**Fig. 1:**
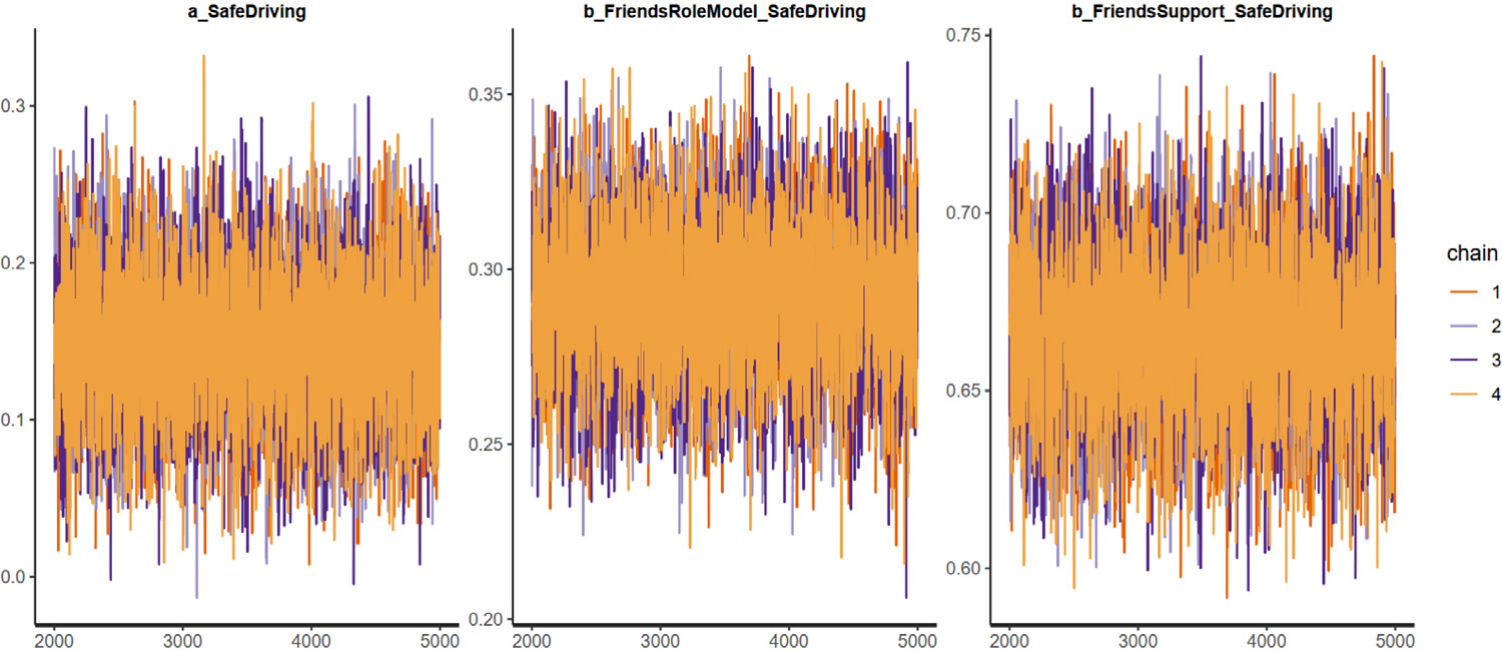
Model 1’s trace plots.

All the estimated results of Model 1 are shown in Table 6. The effective sample size (*n_eff*) is larger than 1000, and the shrink factor (*Rhat*) is equal to 1 in all cases of parameters. These statistics suggest that Model 1’s Markov chains are well-convergent. Visually, the Markov chains shown in the trace plots also fluctuate around a central equilibrium, also confirming the convergence of Model 1 (see Fig. 1). As the Markov chains are convergent, the estimated results are qualified for interpretation.

**Table 6:** Estimated results of Model 1.

Parameters	Mean	Standard deviation	*n_eff*	*Rhat*
*Constant*	0.15	0.04	6650	1
*FriendRoleModel*	0.29	0.02	5415	1
*FriendSupport*	0.67	0.02	4868	1

**Fig. 2:**
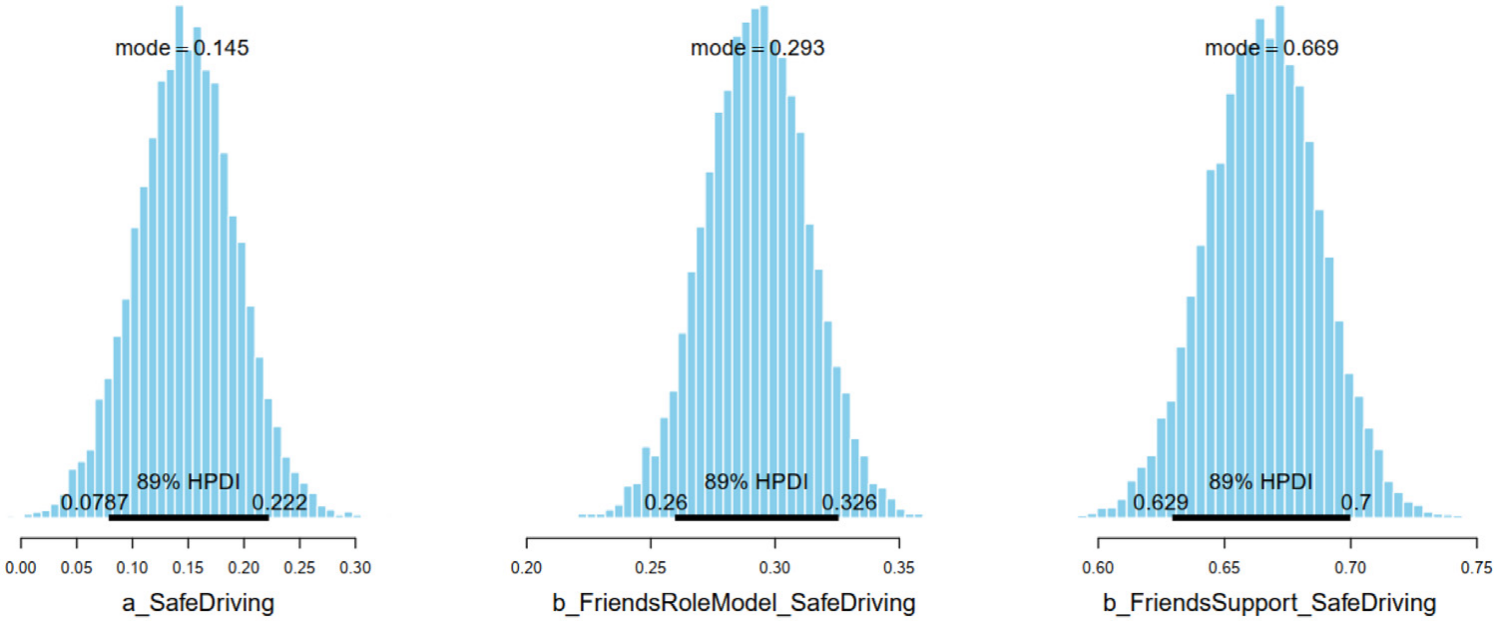
Model 1’s posterior distributions of parameters with HPDI at 89%.

According to the estimated results, *FriendRoleModel* and *FriendSupport* are positive predictors of *SafeDriving* ($M_{\mathrm{Frie}\mathrm{ndRo}\mathrm{leMo}\mathrm{del}}$ = 0.29 and $S_{\mathrm{Frie}\mathrm{ndRo}\mathrm{leMo}\mathrm{del}}$ = 0.02; $M_{\mathrm{Frie}\mathrm{ndSu}\mathrm{pport}}$ = 0.67 and $S_{\mathrm{Frie}\mathrm{ndSu}\mathrm{pport}}$ = 0.02). The posterior distributions with the Highest Posterior Distribution Intervals (HPDI) at 89% of all the parameters are shown in Fig. 2. In all cases, the HPDI is entirely located on the positive side of the *x*-axis (or > 0), indicating that the positive predictions of *FriendRoleModel* and *FriendSupport* are highly reliable.

**Fig. 3:**
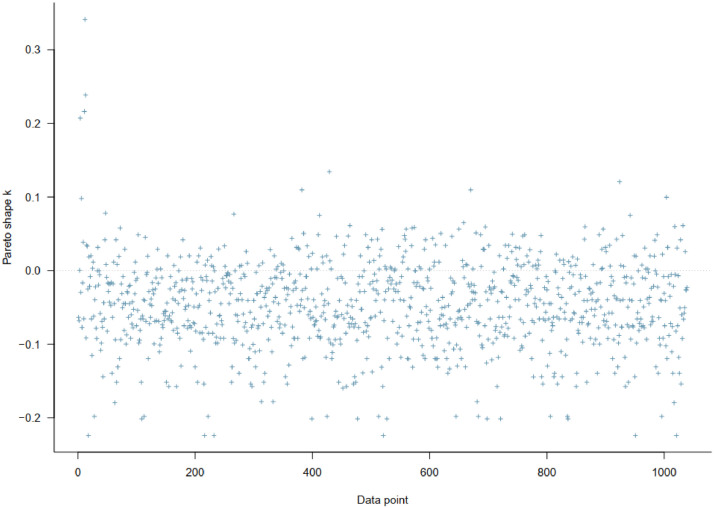
Model 1’s PSIS-LOO test.

To test whether the estimated model fits the data well, we conducted the Pareto-smoothed importance sampling leave-one-out (PSIS-LOO) test [21]. The test provides us with its Pareto *k* diagnostic, which can be used to evaluate the reliability of the estimates. Generally, if the *k* values are larger than 0.7, the model might be deemed misspecified; if the *k* values are lower than 0.5, it indicates a good signal of the goodness of fit between the model and the dataset. As can be seen from Fig. 3, all *k* values are lower than 0.5, implying that the model's goodness-of-fit is acceptable.

Therefore, concluding that the dataset has good construct validity is plausible. All the code snippets used for the data analysis in R are shown below.

**Table:** 

**# Load data and package** data1<-read.csv(“D:/…/ Agressive Driving Data.csv”,header = TRUE,stringsAsFactors = TRUE) library(bayesvl) library(ltm) **# Check internal reliability** Group_1<-data.frame(data1$B1,data1$B2,data1$B3,data1$B4,data1$B5,data1$B6,data1$B7) Group_1<-na.omit(Group_1) cronbach.alpha(Group_1) Group_2<-data.frame(data1$C1,data1$C2,data1$C3,data1$C4,data1$C5,data1$C6) Group_2<-na.omit(Group_2) cronbach.alpha(Group_2) Group_3<-data.frame(data1$C7,data1$C8) Group_3<-na.omit(Group_3) cronbach.alpha(Group_3) **# Prepare data** data1$SafeDriving<-(data1$B1+data1$B2+data1$B3+data1$B4+data1$B5+data1$B6+data1$B7)/7 data1$FriendsSupport<-(data1$C1+data1$C2+data1$C3+data1$C4+data1$C5+data1$C6)/6 data1$FriendsRoleModel<-(data1$C7+data1$C8)/2 keeps <- c(“SafeDriving”,“FriendsRoleModel”,“FriendsSupport”) data1 <- data1[keeps] data1<-na.omit(data1) **# Model construction** model1a<-bayesvl() model1a<-bvl_addNode(model1a,“SafeDriving”,“norm”) model1a<-bvl_addNode(model1a,“FriendsRoleModel”,“norm”) model1a<-bvl_addNode(model1a,“FriendsSupport”,“norm”) model1a<-bvl_addArc(model1a,“FriendsRoleModel”,“SafeDriving”,“slope”) model1a<-bvl_addArc(model1a,“FriendsSupport”,“SafeDriving”,“slope”) **# Generate Stan code** model_string1a<- bvl_model2Stan(model1a) cat(model_string1a) **# Model Fit** model1a<-bvl_modelFit(model1a, data1, warmup = 2000, iter = 5000, chains = 4,cores = 1) **# Visualize logical network of Model 1** bvl_bnPlot(model1a) **# Visualize trace plots of Model 1** bvl_plotTrace(model1a) **# Visualize posterior distributions of Model 1** bvl_plotParams(model1a,row = 1,col = 3) **# PSIS-LOO test and visualization** loo1a<-bvl_stanLoo(model1a) plot(loo1a)

## Ethics Statements

This study was approved by the Institutional Review Board (IRB) of the China University of Political Science and Law on March 18, 2022. The IRB ensures that all ethical standards have been met and that all participants' rights, welfare, and well-being have been fully considered and protected. All collected data have been anonymized and comply with relevant ethical standards and data protection regulations.

## CRediT authorship contribution statement

**Ruining Jin:** Conceptualization, Writing – original draft, Methodology. **Xiao Wang:** Conceptualization, Data curation, Writing – original draft. **Minh-Hoang Nguyen:** Writing – review & editing, Visualization, Investigation. **Viet-Phuong La:** Software, Validation. **Tam-Tri Le:** Writing – review & editing. **Quan-Hoang Vuong:** Conceptualization, Supervision.

## Declaration of Competing Interests

The authors declare that they have no known competing financial interests or personal relationships that could have appeared to influence the work reported in this paper.

## Data Availability

A dataset of Chinese drivers’ driving behaviors and socio-cultural factors related to driving (Original data) (OSF).
